# Supplementary material for: Nucleotide binding by the widespread high-affinity cyclic di-GMP receptor MshEN domain
Source: Nat Commun. 2016 Aug 31;7:12481. doi: 10.1038/ncomms12481 (PMC5013675; doi:10.1038/ncomms12481)
Supplement: Supplementary Information — Supplementary Figures 1-7, Supplementary Tables 1-9 and Supplementary References. [file ncomms12481-s1.pdf]

A) B)

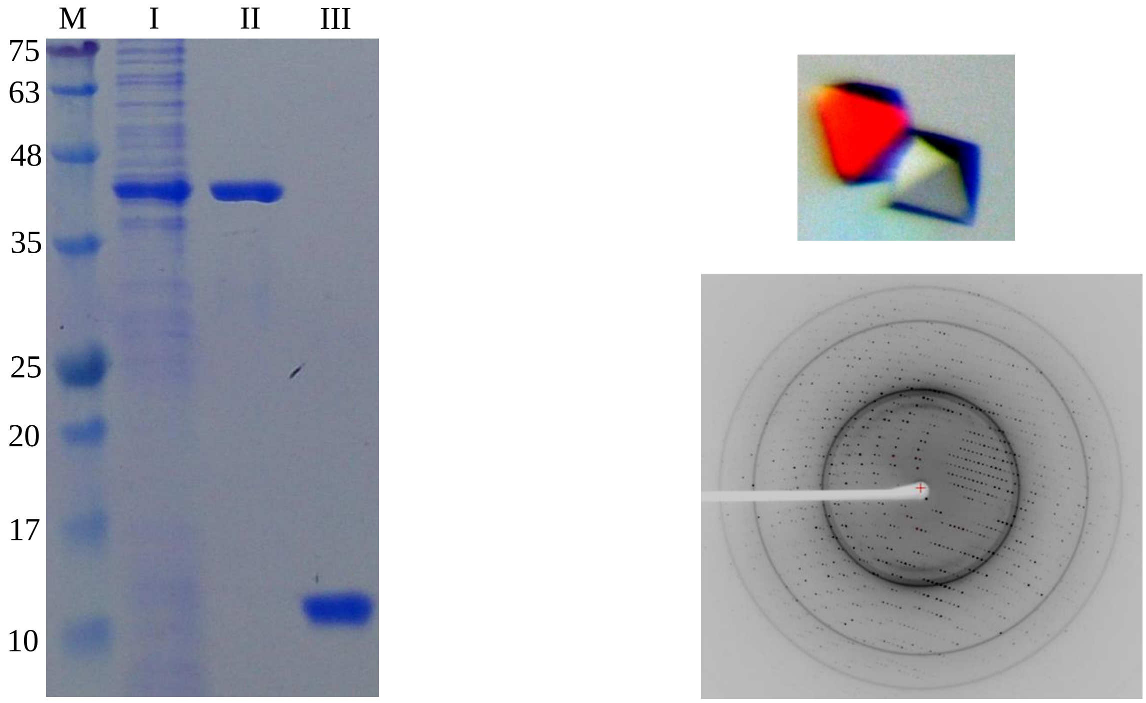

Supplementary Fig. 1: Sample preparation for X-ray diffraction. a) Monitoring of the purity of MshEN samples by SDS-PAGE during sample purification. Lane M: the protein molecular weight standards; Lane I: IPTG-induced cell lysate; Lane II: The GST-MshEN fusion protein after affinity purification; Lane III: purified MshEN after cleavage of GST protein. b) Crystals of the MshEN-c-di-GMP complex and its diffraction pattern reached to 1.37 Å.

Due to the well-resolved electron density map, we could successfully trace the polypeptide chain of *Vc*MshEN from residue Lys5 to Tyr145 without any interruption. Ramachandran plot showed that all torsional angles of *Vc*MshEN-c-di-GMP complex were within the acceptable regions without any outliers. Each *Vc*MshEN structure could be further divided into two subdomains, a four-helix MshEN_N ($\alpha1-\alpha4$) domain ($MshE_{1\text{-}64}$) and an α/β MshEN_C domain (a $\beta1-\beta3$ antiparallel β-sheet surrounded by three helices, $MshE_{78\text{-}145}$), connected by a long linker (Fig. 1a). C-di-GMP was bound mainly in the MshEN_N subdomain.

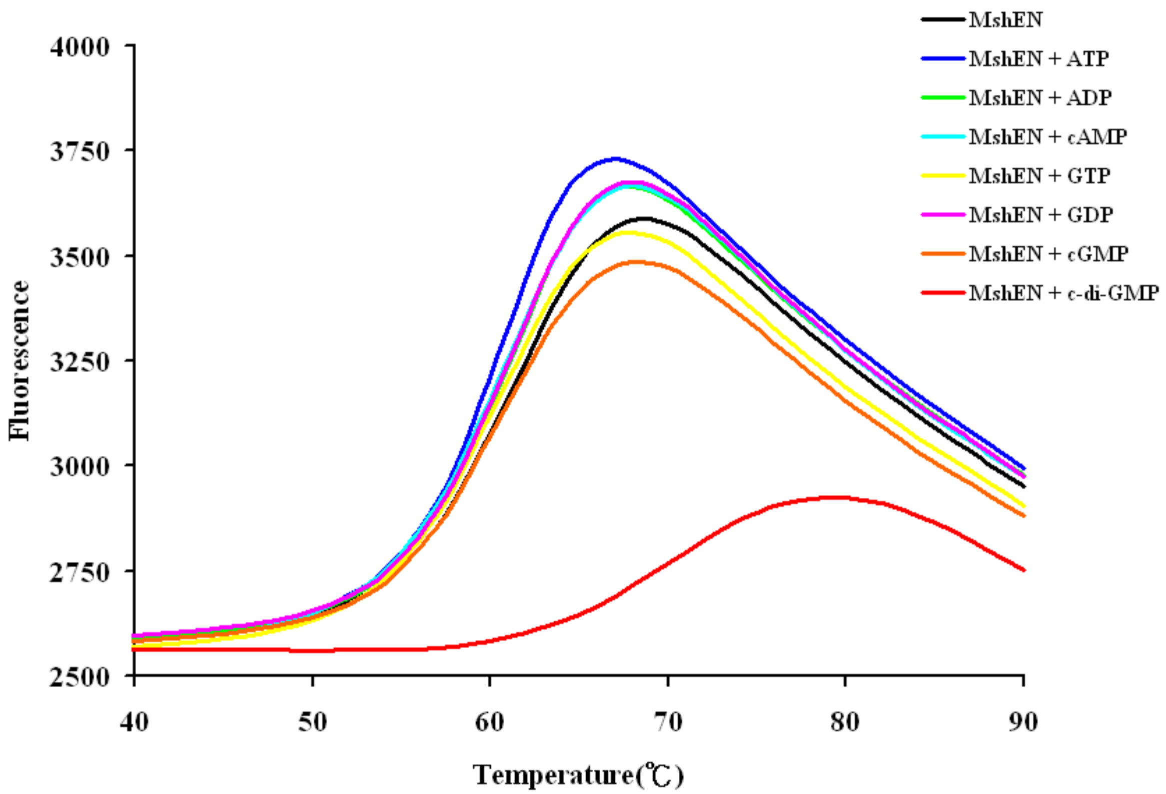

Supplementary Fig. 2: DSF of MshEN in the presence of various adenine and guanine base containing-nucleotides. The DSF experiment was carried out in a buffer comprising 25 mM Tris pH 7.5, 100 mM NaCl, and 0.2 mM $MgCl_2$, and containing some amount of 1:1000 dilution of SYPRO Orange Dye. 10 μM proteins in the presence or absence of 1 mM nucleotides were placed in each well, with the fluoresence monitored when temperature gradually rises. The data clearly indicate that the apo-form of *Vc*MshEN only binds with c-di-GMP with a melting temperature increase of 9° (from 61° to 70°), whereas it does not bind with any other adenine or guanine-containing nucleotides (only 1° or no increase in their melting temperatures).

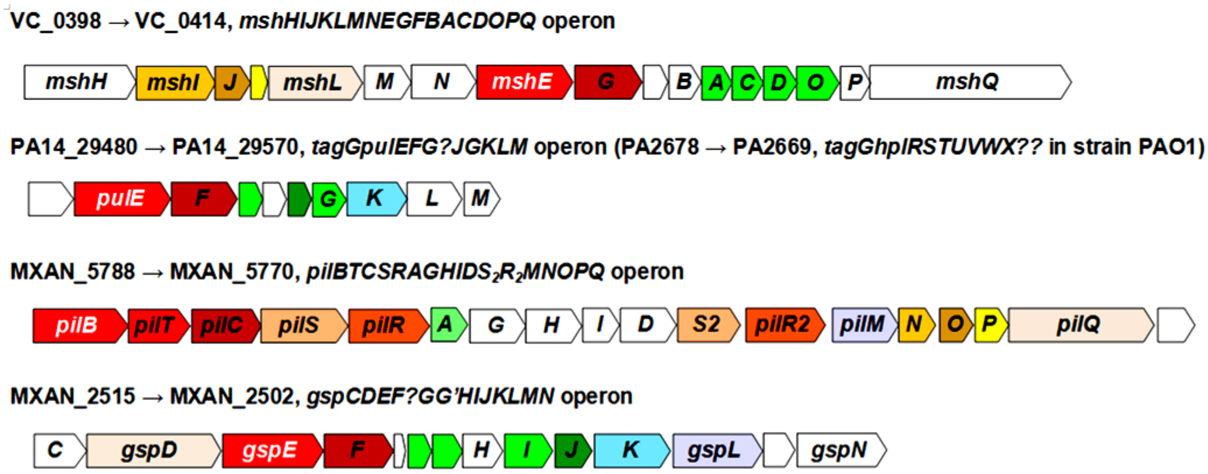

Supplementary Fig. 3a: Operons that encode MshEN-related proteins in model organisms *Vibrio cholerae*, *Pseudomonas aeruginosa* and *Myxococcus xanthus*. Similar genes are indicated by the same colors; MshEN and related MshEN-containing ATPases are in red with white letters. Based on its association with the type II secretion ATPases, the MshEN domain has been previously designated T2SSE_N (PF05157) in the Pfam database[1] and has been listed under similar names (T2SE-Nter, GSPII_E_N, EspE N-terminal domain) in other protein databases. However, the current version of this domain misses the first three helices of the MshEN_N four-helix bundle and, accordingly, does not include the c-di-GMP binding site (Fig. 2). The availability of the structures of the MshEN_N subdomain (PDB: 2D27 and this work) and the MshEN_C subdomain (PDB: 2D27, 2BH1, 4PHT and this work), allows correcting the domain boundaries. Based on the widespread co-occurrence of the MshEN_N and MshEN_C subdomains, and the presence of this domain combination in a variety of domain architectures, including in association with glycosyltransferase, receiver, and other domains (see Supplementary 3b below), we suggest keeping the MshEN designation (pronounced 'mesh-N', which is close to the Russian word for 'target') for this domain combination. For proteins that contain only the MshEN_N subdomain, such *Methylobacillus* sp. EpsG and *Bdellovibrio* Bd2402, we suggest retaining the MshEN_N designation, see Supplementary Table 4. Finally, for the MshEN_C subdomain, which has been previously structurally characterized in type II secretion ATPases from *V. cholerae* and *V. vulnificus* (PDB: 2BH1, 4PHT) and referred to as N1 domain [2], we suggest retaining the T2SSE_N1 designation.

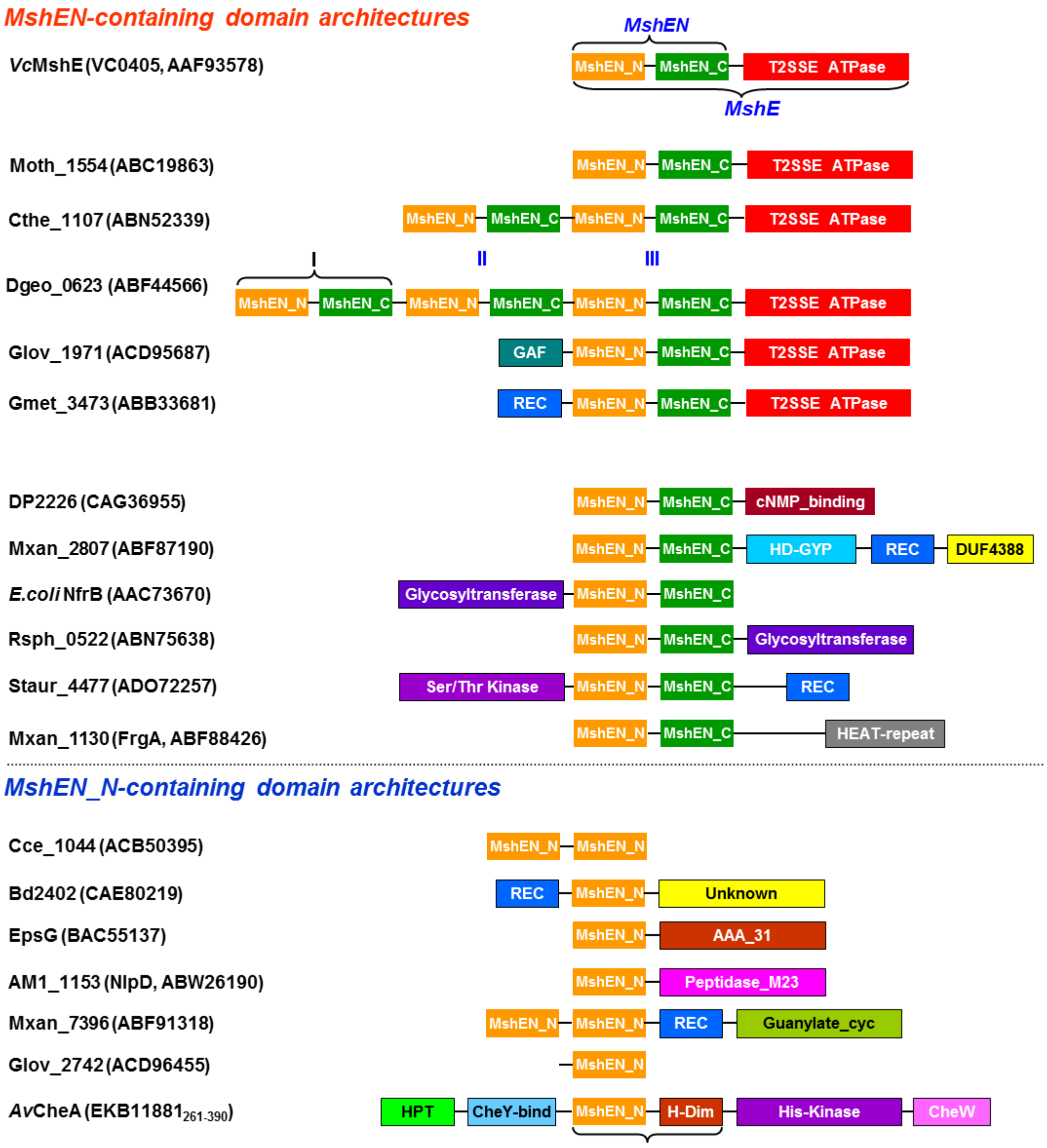

Supplementary Fig. 3b: Examples of MshEN- and MshEN_N-containing domain architectures. The proteins are listed under their locus tags and GenBank accession numbers. Domain names are taken from Pfam and CDD, see Supplementary Table 4 for details. The MshEN_N domains are indicated in orange, and MshEN_C ones in green. Different domains fused with MshEN_N are shown in different colors, the domains are not drawn to scale. Although MshEN_N domains are most commonly associated with the ATPases involved in type II secretion and type IV pili formation (shown in red), domain architectures of other MshEN_N-containing receptors indicates participation of c-di-GMP in bacterial two component signaling (REC, GAF domains), cAMP-mediated signaling (cNMP-binding and guanylate/adenylate cyclase domains), protein phosphorylation (Ser/Thr kinase), polysaccharide secretion (glycosyltransferase), and chemotaxis (CheA and CheX).

Supplementary Fig. 3c: Sequence alignment of the MshEN_N domains and their c-di-GMP-binding sites. The names of experimentally characterized proteins are in bold, names of the protein that have been shown not to bind c-di-GMP are in red. Conserved hydrophobic residues participating in c-di-GMP binding are shaded yellow, those stabilizing the four-helix bundle are shaded gray, branched-chain amino acids (Leu, Ile, Val) are in bold. Panel A below shows sequences mentioned in the text. Panel B shows sequences of MshEN_N domains from model organisms listed in Supplementary Table 3. Panel C shows sequences from Supplementary Table 4. Protein names are linked to the respective entries in the NCBI protein database, organism names are linked to the entries in the NCBI Taxonomy database.

A

```
                          1   5    10   15   20   25   30   35   40   45   50 53
                          RLGxxLLxxGLVxxxxLxxxLxxQxxxxxRLGxxLLxxGLVxxxxLxxxLxxQ
MshE_VIBCH       1 MPINKLRKRLGDLLVEEGIVSEAQLEQALNAQKNTGRRLGDTLISLGFLSETQLLNFLAQQLSLPVIDLSRAHVDIDAV  79
PA14_29490      10 PQVTAEWIPLGQRLLERGLVSGQELERALDLQRRLGGRLGGILVRSGAISENTLMQVLAEQLRLPLVGDDLRKPSEESI  89
Paer_PA3740      2 TMSNQQHSRLGQILINKGLISAAQLDAALQAQLSNHKRLGETLIEQGLLSERQLKKALKKQTNLRLAATLVAALLSPFQ  80
Bbact_Bd2402   206 VDVDDKTTFLGEMLIQSGYATPQDVQEALRVKN--NRRIGDYLIQNNQLSPHAFDLILMEQMNIRLVRTIVDQKIRVNF 282

NFRB_ECOLI     481 TGDTRSLRPLGQILLENQVITEEQLDTALRNRV-EGLRLGGSMLMQGLISAEQLAQALAEQNGVAWESIDAWQIPSSLI 558
EspG_Metsp      14 LKPEKNDANIGKVLLDLGKLTPDEAERVLRLQKQENIRFGEAALRLGFINESDIRQALSVQFEYPYLQPNEGGFSPDLV  92
FrgA_Myxxd       1 -----MPARLAQFLVSRMLLTQERAGEVLRQHQ----TLGGQVDSVLLEQGVASEADVLAMLGEVSGFMPVNLMDFEPN  70
Dgeo_1755      479 EVLQAVRRRLGDQWLFTGALRERELARLLRVQRRAAARLGQLAVQQALVDEAQVRRSLAQTQGLMYLNLTPEMLDHRFL 557
Dgeo_0263        1 MALSIADRRLGAILLEQGYVTDTDLQKALVRHAEVGGRLADILIESGQVGEKRIARAIEEALGIPLVNLLVVTPDAAAL  79
Dgeo_0263_1      1 MALSIADRRLGAILLEQGYVTDTDLQKALVRHAEVGGRLADILIESGQVGEKRIARAIEEALGIPLVNLLVVTPDAAAL  79
Dgeo_0263_2    158 AGSSESGGKLGQRLISRGLINEAQLQVALDVQQQTGEALGHILVTQGLLSEDQLYEVLAEQAGAVYLRNPRDFQPGEEV 256
Dgeo_0263_3    327 QARGGKVKPLGEVIVELGFARAEEIDAALQKQNAGGGRLEDTLVQSGKLSPEMLARSLAAQLGYEYLDPVQNPPDPQVA 405
Tmar_TM0837      1 --MLRRYRKLGEILLEKGFITREELDKALEIQKEERKPLGEVLIETGYITEDQLLEALSEQYGVPILKELPKNIPLNVV  77
Moth_1554        1 ---MDSRRRLGDLLIEAGMLTPAQLEQALQEQKRSGERLGKVLIRLGFITEASMLEVLEFQLGIPKVVLADYHLDPEVV  76
CheA_Aerve     260 ESLPEDSAMLGQLLVNTGALTAEELASGLQAQQA5APKLGEILIAQGAVSEAVVDAALKKQNQTKESKSKEGRYVRVHA 342
PilB_Myxxd       1 -----MSGRLGELLVRENLISVQQLRKAQEEQQKNGTRIGTALVKTGAIEESKLTDFLSKQYGVPAINLKDFDVEPDII  74
PILB_PSEAE       1 MNDSIQLSGLSRQLVQANLLDEKTAVQAQAQAQRNKLSLVTHLVQSKLVSGLALAELSAEQFGIAYCDLNSLDKESFPR  79
PA14_58750       1 MNDTIQLSGLARQLVLHELLDEKAAQQAQSQAQRNKLSLVTYLVQSKLVKGQALIELAADQFGIAYCDLNSLERDSLPK  79
GspE_Xancp       1 MEQRSAETRIVEALLERRRLKDTDLLRARQLQAESGMGLLALLRRLGLVSERDHAETCAEVLGLPLVDARQLGDTPPEM  79
PilB_Vibch       1 ----MLTMNLVAILRQAELISATQEQAVVTQVSASGTSVPEALLELSIFHAQELTEQLSHIFGLPETDLSRYDYANLCQ  75
TIGR02538        1 --------GLGRILVKAGLLTETQAQAALEEAQASGQPLVQYLIQNGLLDPKQLAEFLSREFGVPLLDLNAFDPDALPV  71
Pfam05157        1 ------------------------------------------LVELGLLSEEQLAEALAEQLGLPFVDLEDLPIDPELL  37
COG2804          1 --------------------------------------------------------------GLDELDLYQAQQRRLLL  17
2D27/SEC_STR       -----HHHHHHHHHHH-----HHHHHHHHHHHHHH---HHHHHHH-----HHHHHHHHHHHH-----------------
MshE/SEC_STR       ---------HHHHHHH-----HHHHHHHHHHH------HHHHHHHH----HHHHHHHHHHHH-----------------
```

B

Alignments for Supplementary Table 3

Proteobacteria

***Vibrio cholerae* O1 biovar El Tor str. N16961**

```
VC0405 MshE     1 MPINKLRKRLGDLLVEEGIVSEAQLEQALNAQKNTGRRLGDTLISLGFLSETQLLNFLAQQLSLPVIDLSRAHVDIDAV  79
VC2424 PilB     1 ----MLTMNLVAILRQAELISATQEQAVVTQVSASGTSVPEALLELSIFHAQELTEQLSHIFGLPETDLSRYDYANLCQ  75
VC2732 GspE     1 ----------------------------------------------------------------------MTEMVISPAERQ  12
```

***Pseudomonas aeruginosa* PAO1**

```
PA2677 PulE    10 PQMTAEWIPLGQRLLERGLVSGQELERALdLQRRLGGRLGGILVRSGAISENTLMQVLAEQLRLPLVGDDLRKPSEESI  88
PA3740          2 TMSNQQHSRLGQILINKGLISAAQLDAALQAQLSNHKRLGETLIEQGLLSERQLKKALKKQTNLRLAATLVAALLSPFQ  80
PA4526 PilB     1 MNDSIQLSGLSRQLVQANLLDEKTAVQAQAQAQRNKLSLVTHLVQSKLVSGLALAELSAEQFGIAYCDLNSLDKESFPR  79
PA5210 PulE     6 TAAQDRLLDLGDLLRELVAQGRVGQEQAEQCLAIRRSAVRNQQHPLEFLAAQQVEDLKRPGRKLDLETLTQWLAEYAGQ  84
```

***Bdellovibrio bacteriovorus* HD100**

```
Bd1509          1 ----MSSLKIGEILVKQGLLKPDQLALAMEEQKKTGQKLTNSIIQLGYLKDNQILRAVEKHFAVPGVEVNTFEIDATVI  75
Bd1596          1 ----MASVDVSTILAKSTSLTQDQIRSVLNNPSVV2VTVGEALAAKEFSTADEVVSDLCKELGLDFIRDIPVSEIAVDL  76
Bd2402        206 VDVDDKTTFLGEMLIQSGYATPQDVQEALRVKNN--RRIGDYLIQNNQLSPHAFDLILMEQMNIRLVRTIVDQKIRVNF 282
```

***Escherichia coli* str. K-12 substr. MG1655**

```
NFRB_ECOLI    481 TGDTRSLRPLGQILLENQVITEEQLDTALRNRVE-GLRLGGSMLMQGLISAEQLAQALAEQNGVAWESIDAWQIPSSLI 558
```

***Methylobacillus flagellatus* KT**

```
Mfla_2024      19 LVNEFSEFTIGSLLLRMGKITPEDAERIMRLHKEKGLRFGEAAQALGLITEDDIQQVLSRQFNYPYLSPDQNSYLNELV  97
```

***Myxococcus xanthus* DK 1622**

```
MXAN_1090       1 -MASPSRNRIGDILVKARVIDDLQLRSALATHDQWGGRLSRIIADLGLATDDVITEAICQGLGMQRIQLGNVTRDAGAL  77
MXAN_1130       1 -----MPARLAQFLVSRMLLTQERAGEVLRQHQTLGGQVDSVLLEQGVASEADVLAMLGEVSGFMPVNLMDFEPNPEVA  74
MXAN_1145      58 CQSNLLHETLGKYLVEKGKLSETDYQKALAESISAEVPLSGLLVQKGLISPFDLYKQLQANLAHKLLDCFRWVDAKYRL 136
MXAN_2513      31 GQAYLCGRPLGEILRA2PSLTEEKLQEALAIQDEKGQRIGEALVGMKAVSEEDVAKALGHQLDLPYLARIFAEEVDAEL 110
MXAN_2658      15 SRDDFTTLFVLEALVAQGLLQPQQAQEVLAREGAARARVLKAHQGATGGSKEAARYDVSPVEIVAAFQVPLSNGRGVLD  93
MXAN_2807       1 -----MVKRLGERLIEAGLVNAGAVEQALEHQKITGHKVGDCLVELGLLQEAALLRFLAAEFQTRFVSADKLAKARIAT  74
MXAN_2924       1 -----MAEKLGAILVRKGLITQAQLDEALRAQLIYGGRLGSNLVELDILDIDTLAMVLGEMCRYPVAQEADFVAAPDAV  74
MXAN_3211      50 ASSNQPREFFGQFLINMGHLTEDQLEKAFSTQAQtRIFLGKILVMTGLVPEATVRGTLSHKFREMILDAFHWEDGDFLF 128
MXAN_3836      63 VSLPGRKRRLGEILMDAGLLSETQLRSALAEQRKWGGKLGLTLVQMGYVDESSMVHALSRQLAIPTVDLEQHAASAVVL 141
```

```
MXAN_4436     1 -------MRLGEQLLKDGLVTAEGLEEALEAQVVHGGRLGTNLVELGLLSEVDLAKALGKVHNSAFASGEMVPDPKAME  72
MXAN_4438   259 AASNLAAERFGRFCVRQGVLPEARLAEVAAFAKERGLRTGEAMLRMGLMDAAQREQLLVEQVKEIIWSTFTWKEGGYGF 337
MXAN_4627    16 DEFLQQLSRGGELLAAGRVHEAQPFLERAHQLHP5QNLLGLCYFKLGLYDRAAELYEMLVRDNPVDPTLRVNLGLVYLK  98
MXAN_4666     1 -----MRKKIGELLVEAGVVTEEQVRVALGRRGA2SHRLGEVLVAQGLCTPTHIAQALSAQHALPFVALPEEIPANVAG  75
MXAN_4667     1 ----MAQIKLGELLIKANVLQESQLKAALAEQAKWGGKLGEILVRMSLVSEDILVRALSKQLGMPAVNLDAVQMVQPHV  75
MXAN_4739    51 SVTRKRKELIGAMLVRSELITETQLEAALETQRRTLKRLGDVLVSSQALTAERFQAMMQLQATETLYRLFTWKSGTYEF 129
MXAN_5196   142 ASSDDPADRLGEVLVRMGYVERAQVEAALREQP--PSKVGRSLVEKGLLKSHDLFKCVTHQVSEIFHAIVLCREGTFFL 218
MXAN_5497     7 LMETGSRRPLGEILLEQGVLNRAQLRVGLVHHHEVHVPLGRALVREGLCSGADVLRGLAEQFGVDAVDLERTPPDSRRL  85
MXAN_5788     1 -----MSGRLGELLVRENLISVQQLRKAQEEQQKNGTRIGTALVKTGAIEESKLTDFLSKQYGVPAINLKDFDVEPDII  74
MXAN_6627   285 ALSNLLADRFGQFLVRVGKIKPEQLQDASAVAAQSNRRTGDVLVERGLLKDTERLYYVGQQVKAVIYSLFAWDEGTYVL 363
MXAN_6863     1 ----MARKRIGELLLEQRAISVAQLEAGLAAHRKSGQRLGATLIAQGAITEATLADALSQALGLPRVDLAATTPEWAAV  75
MXAN_7396    10 PTMSASSPLFGDLLLKLGIVSPGQVQEALALQALTGQRVGEALISLGYVTREQIQDALGEALGLLHQDKSPL-------  80
MXAN_7396    81 ------QPALGELVGLKYVTLAQLDEALARQRRDGRKLGEILVELGHCTYKQIYEALGLQNRIAGRQDLPRPSSDGRR 163
MXAN_7500     1 -----MKKRLGDILLERGVVDALQLHSALAYQRKWGVPLGQVVVDQRFSTTQQVLEALAFQAGMQTVDLDVQPPDASLT  74
```

***Rhodobacter sphaeroides* ATCC 17029**

```
Rsph_0522    24 PSPAEAAEPLGVMLLREGHLAPHRIMAALSHGGRPSAPLADVLLAEGALSEDEILAMMARRSGLPVLDPAAERPDPRLI 102
```

***Shewanella oneidensis* MR-1**

```
SO_0416 PilB    1 MPTTGLHLGLSTLFIRKGLLNEEQMATAITKSRQNKQTLVTTLVQTKLVSARSIAELCYEEYGTPLLDLAEFDVSAIPE  79
SO_4109 MshE    1 -MKPRLKMRLGDLLVQESIITEEQLQQALGEQRKTGHKLGRTLIDLRSITETQLLQFLSQQLNLPLLDISKRSIPSEVV  78
```

***Stigmatella aurantiaca* DW4/3-1**

```
STAUR_0316     1 --MSSSSPLFGDLLLKLGIVTPSQVQEALALQPRTGQRVGEALISLGYVTRAQLHDALSEALGLNHDKG-------   67
STAUR_0316    68 ----PAHPPLGELVGLKYITLGQLEEALAFQRKDGRKLGEILVEMGHCTYRQIYEALSLQGRITGRQEAPRQVLQ  139
STAUR_0993     1 ----MERKRIGEILLQRGAISPVQLEEGLKAQRQTQQRLGTTLVAQGAITEATLVQALSEALGLPVVDLEAIAPDW   72
STAUR_1269   256 ALSNLLADRFGQFLVRVGKIRPEQLQDATGVATQSQRRTGDVLVERGLLKDTERLYYVGQQVKAIIYSLFSWDEGT  331
STAUR_1456     1 -------MRLGEWLVHNGALTPEQVETALAYQNRWRCKFGQAVLELNMIPREPFLRLLAGHLKVAFIRPEQIDKVP   69
STAUR_1649     1 -MAAPQRNRIGELLIKARVIDDLQLRSALAQHDQWGGRLSRVIVDMGIANEETIINAICQGTGMQRMHLGHVTRDP   75
STAUR_1674   197 VDSSHAEDALGTVLVQARLLTPEQIQQAEAARPRFGGDLLVALFSLGLLQPATAFTQLSQRALSILHKGLRAESGT  272
STAUR_1731    58 CVSNLLHETLGKFLVEKGKLGEADYQKALSESIQTGQAIGTLLVQKGLLSPFDLYKQLQANLGLSLLDCFRWTDAR  133
STAUR_3194    30 SQAYLSGRPLGEILQATASLSEEKLQEALGVQAEKGGRIGEVLVGLKAVSEEDVAKALGTQLDLPFLQRIFVDEVD  105
STAUR_3645    50 ASSNQPREFLGQFLINMGHLTEDQLGRAFETQRVTDMLLGKILVMQGIIPEPTVQNTLNLKFREMLLDAFQWGEGE  125
STAUR_4365     7 SPPPFRKKRLGEILLDAALLSETQLRTALAEQRKWGGKLGHTLVQMGFVDENSMVHALSRQLQIPSVDLAQVAPPA   82
STAUR_4477   394 GRQPRAGRRIGDILVARGMLPPEALEQALTLQKRLGGKLGQVLVGERLLEAEELVRALSEQSGMPHISGERLQTMP  469
STAUR_4548     1 -METGTRRMLGEILLERGVLSRAQLRLGLVHHHEVRVPLGRALIRERLCTESDVLQALSDQLGIGAVALERERLDP   75
STAUR_4792     1 -------MRLGELLIQEKLITRQGLEEALESQVVHGGRLGTNLLELGLLSEKDLA-RLLGQLHGCAHASGELTPEP   68
```

```
STAUR_5176      1 -----MAEKLGALLVRKGLITQTQLDEGLKAQMIYGGRLGTNLVELEFLDIEKLGEVLSEQSRYPQATIQEFEAVT   71
STAUR_5245      1 -----MAKRLGERLIEAGLVTAEAVDKALDHQKITGHKLGDCLVELGLLPEAALLRFLATEFQTRFVSADKLAKAK   71
STAUR_5447      1 -----MRKKIGELLVESGAVTEAQVRTAMAQKRNY3HRLGSVMVAMGLITPTQLARCLAVQFDMPFVELPEIPPQV   73
STAUR_5448      1 ----MAQIKLGELLIKANVLQESQLKAALAEQAKWGGKLGEILVRMSLVSEDILVRALSKQLNIPAVNLDAVQMIP   72
STAUR_5589     51 SITRKKKDLIGNMLVRAEIITETQLEDSLETQRRTLKRLGDVLVSSGAITADRFKKMMQLQATETLYRLFSWDAGT  126
STAUR_5733    107 ATSTERYERLGAVIVQLGLITSEQLTHAIGLVTPS-RRLGQVLVTEGTVSESNLYNAMTFLVREVVLNLFEMMEGN  181
STAUR_6393      1 ----MRKKRLGDLLQAAGLVDELQLRAALGFHHKWGTPLGQVVVDLGFCTAQQVLELLANQAQLPMVDLDAEMLDP   72
STAUR_6458      1 -----MSGRLGELLVRENLITVQALRKAQEEQQKSGTRIGTALIKTGAIEESKLTDFLSKQYGVPAINLKDFDVDP   71
STAUR_7531    441 RARPTVRRQLGDLLIAAGKLSEAQLHAMLERQRRDGGKLGEWLVAEGLVTDEDVVAAISEQLGIPFIAEHQLRHLP  516
```

***Xanthomonas campestris* pv. *campestris* str. ATCC 33913**

```
XCC0660 XpsE  13 IEQRSAETRIVEALLERRRLKDTDLLRARQLQAES8GRLGLVSERDHAETCAEVLGLPLVDARQLGDTPPEMLPEVQGL 100
XCC0678 NrfB  14 PDIGRERGLLGRSLVSAGVITDEQLRAALALQQRWNSRLGDVILAQRGVPAQRFYAIVAAHFGLQFVDLVQQPPDPELL   92
XCC3097 PilB   1 MSVVLTANLVGITGIARRLVQDGALEEAVARGAMDQAALAKVPLPQWFAEKKLVSAAQLASANAVEFGMPLMDVSVFDA   79
XCC4088 GpsE  10 STSAPLPLPRGRLMFDPVAAALLADGMVVPHEHERVQFSAAGVRNASEVHPLVLLANLKLHAAQPPAGELGERLTEWL   86
```

Other taxa

***Acaryochloris marina* MBIC11017**

```
AM1_1153         1 ----MYLVRIGKVLTQKGLISDFQLQQALQIQKQTGQKLGEILIQQGHISQLQLKQVLVEQRCRNFLACSLLTLSTLGP   75
AM1_2467 GspE    9 ELVRNNFSPSNEKLIKSGYVDQARLREAIYES----RRKGRSLLA2QKMTGKPLPPRLLRQIKRQQLFELKILYGLDCL   85
AM1_0419 PilB   11 LVTRHNFSPFGNKLISTGYVDQDQMREATLES----RRSGRSLVE2QGLTGQSLPPELLRQYKRQQLFELKILYGIDSL   86
AM1_0779       264 IPVNTNLKPLGCYLLEANLVTHAQLEEVLQEQQSDHKPIGNLLAEKGWIHQNTVEYMMKNIIQPHRKYQQQASIINVG- 341
AM1_1361       211 ATHSPDSKRIGGYLIEAGLLSPAQVEVVLSDQELTGMRFGEILVSRGWLKSQTIEFLFQNVILPHRTLAKKALETARKE 289
AM1_1657       750 TEQLTHSQGIGAHLLEAGLLTSEQLEVALEDQERQ2ARLGEVIQKRGWVKERTIQFLLNQVNNTLVDHPALNACTQLGN 829
AM1_1845         2 LSYDPLTKRLGSYLIDAGLLSESQVDVALNDQEATGMRFGDILVERGWIKRQTIEYLTRKIIRMEREIGEPLKSGLIET   80
AM1_2351         7 SRSSQPYQPIGAYLVEAGLLTDAQVGVALADQNVTAMPFGEIVVARGWVKEQTIEFIMHRVVLPERSIQAASSGMQDCL   85
AM1_4892       200 QKNFADSKKLGNYLMEAGLLTSAQIEVALADQQITGMRLGEVLVRRGWVKEETIEYLMQKVILPERTSSQDQSTSYMEL 278
AM1_4963         1 -MPSASTKPLGAVLQQAGLISVAQVSLALQEQTINQHRLGDLLCEHGWLKEPTADFFAEQWPTLFHDPDPLPLEQYLRQ   78
AM1_6001         5 PTLIQNSHKIGERLCASDLITPHQLETALYEQRLY2MRLGEILTLHGWVQQQTADFFGDQWPYPDTQAEPVPIGSYFRR   84
AM1_6196       179 ASRYPGIKRIGSYLVDAGLLSLAQVEVVLSDQDSTEMRFGEILVSRGWLKFETVEFVFQNVILPQRTLAKKTVDAAYPS 257
AM1_B0148       10 LLVRNNFSPSNEKLIKSGYVDQTRLREAIYES----RRKGRSLLA2QKMTGKPLPPRLLRQIKRQQLFELKILYGLECL   86
AM1_F0070       10 LLVRNNFSPSNEKLIKSGYVDQARLREAIYES----RRKGRSLLV2QKMTGKPLPPRLLRQIKRQQLFELKILYGLECL   86
AM1_H0057      214 ASRYPGIKRIGSYLVDAGLLSLAQVEVVLSDQDSTEMRFGEILVSRGWLKFETVEFVFQNVILPQRTLAKKTVDAAYPS 292
```

***Aquifex aeolicus* VF5**

```
aq_1971          7 EKLLTLFAKAGYIKDGDIKKVLSEIQKDEDPIEA-LIRLGLITEDQLTEFFKQHIPSRVVEEINTSEIPAEVLNIVPRN   84
```

Cyanothece sp. ATCC 51142

```
cce_1044_1      1 MVTANSKQPLGTILQEAKLITPYQVETALNEQKK2QRRLGEILAEKGWIKQQTADFFAEEWEKVLTQAQ----------  70
cce_1044_2     71 ---QGTPQSLGYYLREAGLIDDYQLDDILAEQGQ3WMRIGALAVLKGWLNQTTVDFLLTHLHPDKAGDSPFIRAKQ--- 145
```

Deinococcus radiodurans R1

```
DR_1964       -41 LALSIGDRRLGAILLDQGYLGDNDLQRALERHSEVGGRLADVLIDSGMVGEKRIARAIEEALGIPLVNLLAVQPDPAALR  39
DR_1964       117 SDAGRTGGKLGERLITHGYITDAQLQVALDAQQQTGEALGATLISQRAITEDQLYEVLAEQEGTTFLPNPSGFHPGEEVL 197
DR_1964       286 QAREGKVKPLGEVITELGFASPDEVDSALQKQNVGGGRLEDTLVQSGKLSPEMLARSLAAQLGYEFLDPIQNPPDPKVAL 365
```

Moorella thermoacetica ATCC 39073

```
Moth_1554       1 ---MDSRRRLGDLLIEAGMLTPAQLEQALQEQKRSGERLGKVLIRLGFITEASMLEVLEFQLGIPKVVLADYHLDPEVV  76
```

Nostoc sp. PCC 7120

```
all2444        13 LTTRTEFSPFGNKLVQSGYINTEQMRQALIESR----KSGRPLTEVLESITGRQLSPEYLRQYKKQQLFELKILYGVEY  87
```

(Pepto)clostridium difficile 630

```
CD3512          1 ---MAKKVRIGDKLVEKGYITEEQLKWALSEQKNSGKRLGEFLVQEGLIDSNLLISVLKELLDIESIFLEGTEIDTLAT  74
```

(Rumini)clostridium thermocellum ATCC 27405

```
Cthe_0853       1 -MQKQKRKGLGDILVEAGLISKEQLDKALKLQKKTGQKLGVLLVSEGIVTQEDIMRVLEEKIGVLRVALEECNIDpAVC  78
Cthe_1107       1 -MLGVDTRGIDEILLEMGVLKIVDLKKAWDIQRESNKNIEDVLLELGLVSQKDIMHANAVKMGIPFVDLSTYQIsDSSV  78
Cthe_1107     208 NESGIFKDKIGNILVRAGVITQDQLENALSIQKKSGGLIGQILVKQGYIDRRSLYEFLQKQMGVEYVDIeGIEIDEDII 286
```

Rhodopirellula baltica SH 1

```
RB12774        22 RSSTMAPRRIGQILVDLGFLTDDQLQIVLDEQEQ2GALFGKVAEDMQLVTDEQLIQALAEQMGMQTVSLEDAKLEPEVM 109
```

Synechocystis sp. PCC 6803

```
Slr0063        13 VALRNYFSPFGNKLVAGGHVDAEQLRQALVQV----KKTGRSLPE2KAVTGRELSPELLRQYKKNQLFELKVLYGVDSV  88
Slr0079         6 DSPEFSLSPFEQALIEAGYLSLSQIHQALMEA----RRTGIALPT2ETILGESLPDELHRQYQAQQRFALSVIHGVKFF  81
Sll0547         1 --MVSSQKPIGIILQEAKLITPGQLEVALYEQKQFQMLLGEILSHHGWISQETADFFVDLWPAMVGQP-----------  66
Sll0547        67 -----VQGPIGYYLKMAHLLDEDQIERILREQRRLGVRFGSVAVLKGWLKEETINFFVKNIVREAQEDTPSAALVLPVP 140
Sll1921         1 MAQSPCSRLLGEILREADLITEAQIQVALQDQQYSPLPIGEILVVRGWLHQETVDFFAESWPRMVKTRDRRPLGYYLQQ  79
```

Thermotoga maritima MSB8

```
TM0837          1 --MLRRYRKLGEILLEKGFITREELDKALEIQKEERKPLGEVLIETGYITEDQLLEALSEQYGVPILKELPKNIPLNVV  77
```

***Thermus thermophilus* HB27**

```
TTC1622       2 SVLTIGDKRLGAALLDAGLLTDEELQRALERHREVGGSLAEVLVDMGLLSERRIAQTIEDRFGIPLVELHRVEIPPKVK  80
TTC1622     159 SGEGQKDLKLGELLLQKGWISREALEEALVEQEKTGDLLGRILVRKGLPE-EALYRALAEQKGLEFLESTEGIVpdPSA 236
TTC1622     327 QKGLPRAKPLGEILVELGLARPEDVEEALQkQRRGGGRLEDTLVQSGKLRPEALAQAVATQLGYPYVDPEEDPPDPGAP 405
TTC1844       1 ----MARPRLGELLLDLGYITEDQLKAALEEQERTGDLLGQILLRRGYIKEQDLVRALADQQRAPLIHPAQTPLDPQAL  75
```

C

Alignments for Supplementary Table 4

```
MBAV_005423   1 -MNIGKKNKLGTILLNRGLITQEQLDSALQRQKVSRNRLGVILIKMGYVTEADIVEAFSRQSDITRIDLSTAKPNAEAL  78
MBAV_005423 142 FYTGVERPRLGHILLQTHVISEEQLQKALHLQRTNGKSLGNILVDMAVVTEEDILRTYSVQMGFPYVDLSLIKVDQRAL 220
MBAV_005423 307 PPGQATSVKLINLFLNSNMITQSDLDIALAEQKNTRRNICSILLDLGMVRDRDIARTWALYYNVPYQNLTGVEIKPDAL 385
MBAV_005423 449 HYYGLPFKRLGDILLESNLISHEDLTVCLKKQKEA3CRLGEAIIKEGLVAEDHVYKAFARQLNIDYSDILNESPSADVI 529
Glov_1971   189 TTKIIRQRSRYNYLLDRNILDDKSIEKASVHPDV--AKFGLDAVLMREYKVPREEMAKALSLFFGTEFIKYEPAAPAME 264
Gmet_3473   183 GLFDEDKSMLGSILTEAGVIRKDDFAAVLQGK-----KPGELLVDALVRTSVSTEAKILKTLQNHLGVEFIDLREANIT 256
DP2226       28 DQSGAGKIKLGELLSKAGQITTTQLETAKKLVKR-GQRMSLVLRQLEYIDEGTVFNFLSRQHNYPAVVIEKEPPTAKVV  97
Rsph_0522    13 PSPAEAAEPLGVMLLREGHLAPHRIMAALSHGGRPSAPLADVLLAEGALSEDEILAMMARRSGLPVLDPAAERPDPRLI 102
Glov_2262     1 ---MTAKKLLGEILVNKGILSPLTVERMIALANREQKRFGWFLEDKGLITGHELSAALAEQFNMKhLTSIEQYSYPKEL  76
Glov_2742     1 ----MAVKFFGQFLVEQDAVTGESLLHAIELQERTNLKLGEMAVAMGFITQQDIETAHSAQLSK---------------  60
Glov_2742    61 ------DMKLGDLLVELGFLSPRQLEEVIAHQKATHLYIGEALVKVGALTPEKLDHYLAAFKADQAPYVANHVELPAWL 119
DVU_1594    245 LEQDGAYKRLGEILLERGDVATEDLRRVLEEQR----PIGELLAEAGIVTPDQVASALAEQKTVRELRRSRTEDGDKPA 319
```

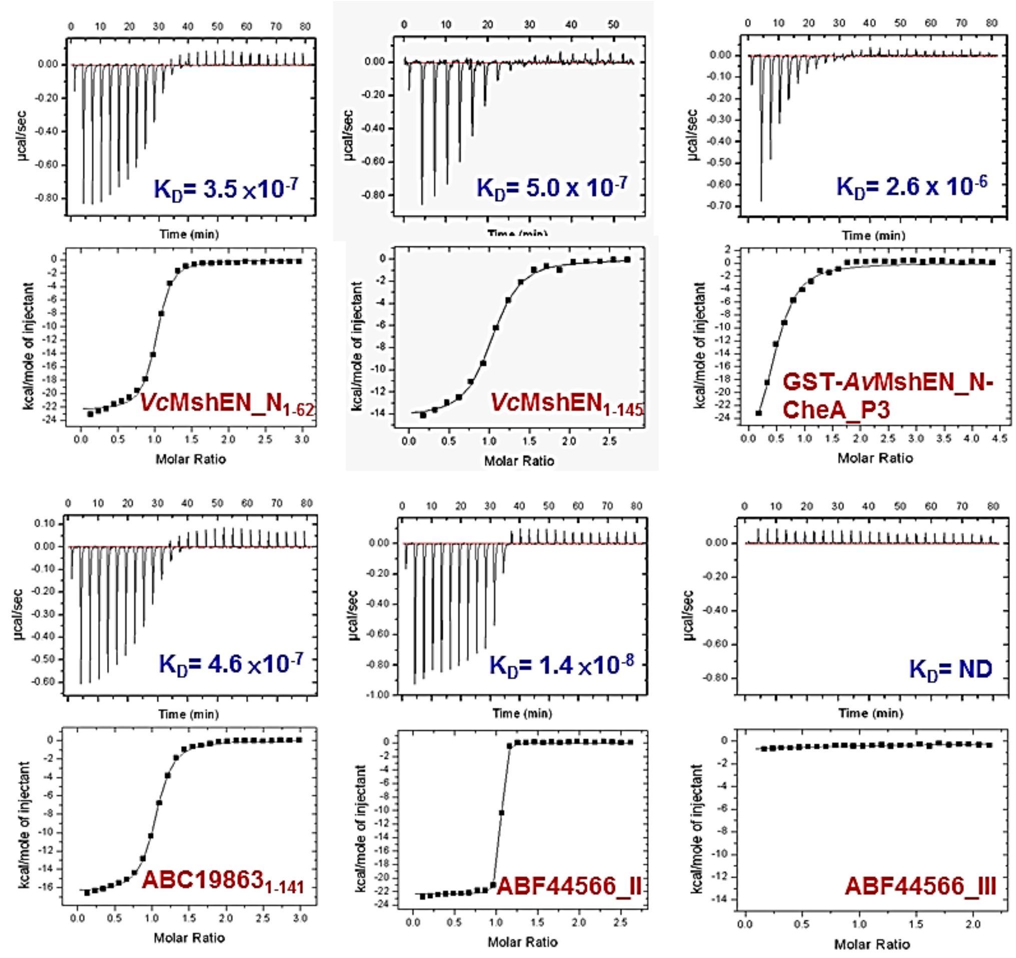

Supplementary Fig. 4: Selected ITC data of some samples listed in the Supplementary Fig. 3.

The MshEN_N domain comprises 53 residues, with more than ten conserved residues. Some of them are absolutely required, while others are more tolerant of change. The binding constant of the *Vc*MshEN_N$_{1-62}$ domain is even stronger than *Vc*MshEN$_{1-145}$, consistent with the structural data that c-di-GMP is mainly located in the N-terminal domain of MshEN. ABC19863$_{1-141}$ has a similar binding constant with *Vc*MshEN$_{1-145,}$ since only several hydrophobic residues are changed to other hydrophobic residues, such as I19M, L49I, and L54M, which are interchangeable. However, it is important to note that while ABF44566_II has a rather strong binding constant (1.4 x 10^{-8} M), its homolog ABF44566_III has completely lost it binding affinity with c-di-GMP. Sequence comparison (Fig. 2a) indicates that ABF44566_III lost the binding due to some critical residue changes. For example, G40E and F48K are possibly the two most crucial changes. On the contrary, *Av*MshEN_N-CheA_P3$_{261-390}$, which contains an MshEN_N domain fused to a CheA-P3 dimerization domain, exhibits a binding constant of 2.6 x 10^{-6} M. Although it has four residue changes in the MshEN_N domain, namely, R1M, I11A, R30K, and L40A (Supplementary Fig. 3c-A), these changes are tolerable and only weaken the c-di-GMP binding somewhat.

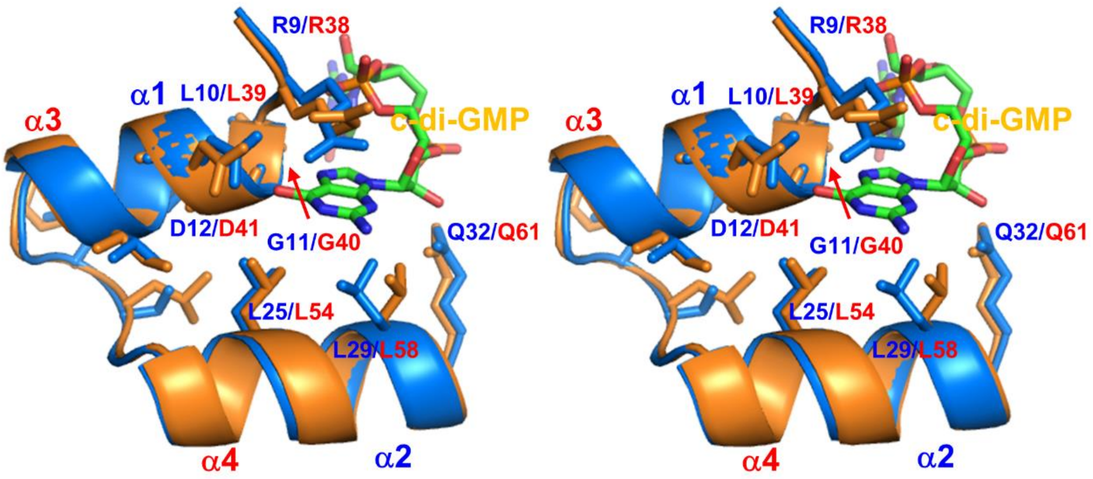

Supplementary Fig. 5: Superimposition of the Gua1 and Gua2 binding sites. Residues from Arg9-Gln32 of the Gua1 binding residues are annotated in blue, while the Arg38-Gln61 of the Gua2 binding residues annotated in red. The red arrow indicates the region of the H-bonding between the Gly11-Asp12 or Gly40-Asp41 and the Hoogsteen base-edge of Gua1 and Gua2, respectively. Amazingly, although the two guanine bases are located in a different environment (Fig. 2a and 2c), the Cα' atoms of the conserved binding residues align very well, with a RMSD of only 0.8 Å over all 25 residues. The α1-linker-α2 segment almost superimposes entirely with the α3-linker-α4 segment, with most of the side chain atoms of the conserved residues including Leu10/Gly11/Asp12/Leu25/Gln32 positioned almost on top of each other with Leu39/Gly40/Asp41/Leu54/Gln61. Only the side chains of the Arg9/Arg38 and Leu29/Leu58 residues exhibit some differences**.**

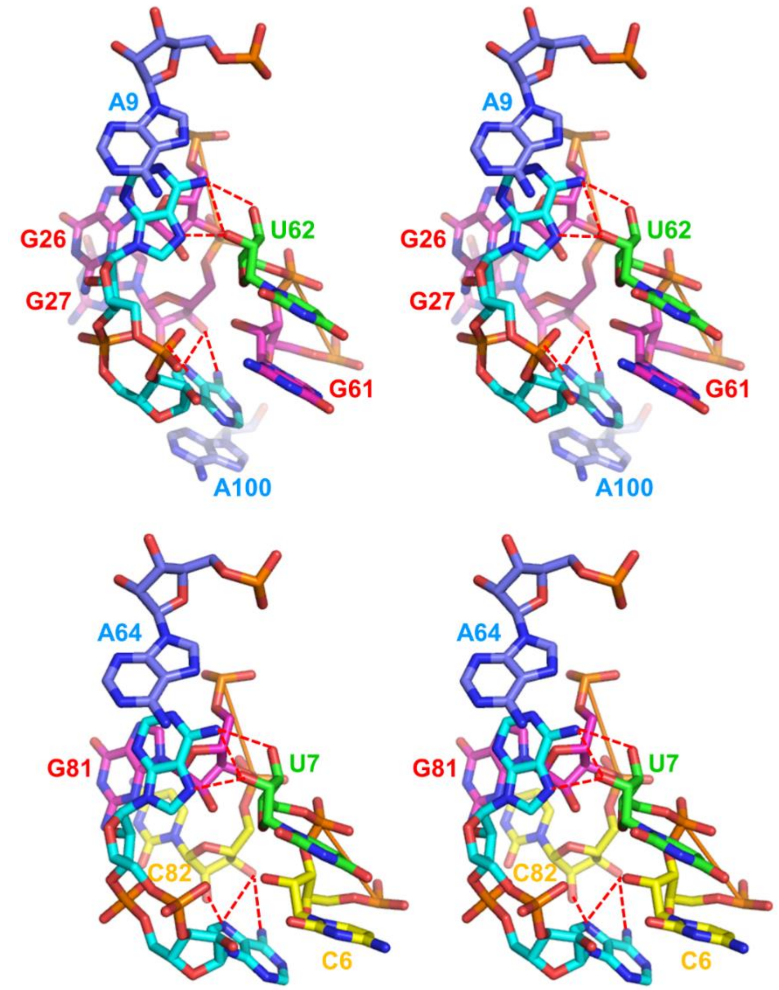

Supplementary Fig. 6: Recognition of the two c-di-AMP ligands in the yadO-riboswitch (PDB: 4W92). This figure was drawn with the c-di-AMP in a similar orientation to that of c-di-GMP in Fig. 3a. The carbon atoms of c-di-AMP are drawn in cyan, and those of adenosine in marine blue, guanosine in red, uridine in green and cytosine in yellow. Each adenine base is sandwiched by an adenine from one side and by the ribose from the other side. Its Hoogsteen-edge H-bonds form three H-bonds with a uridine or guanidine (a) or a uridine or a cytidine (b) that are drawn in dotted red lines). However, the binding characteristics are rather distinct. In the protein-binding mode (shown in Fig. 3a), the first two helices of the four-helical bundle (α1-linker-α2) form a similar guanine binding environment with the last two helices (α3-linker-α4) to form a pseudo-dimer for binding Gua1 and Gua2 bases of the single c-di-GMP ligand (Supplementary Fig. 5). In contrast, in the RNA-based c-di-AMP riboswitches, each forms a pseudo-dimer to bind two c-di-AMPs. But unlike the stacking of the guanine bases with the Arg side chain atoms and the surrounding tri-Leu hydrophobic cluster in the MshEN_N-c-di-GMP complex, the adenine base of c-di-AMP in this riboswitch is stacked by an adenine base (A9 or A100) in one side, and by a ribose of G81 or G61 from the other side (Fig. S6a). The binding scheme of the second c-di-AMP ligand is similar to that of the first ligand and is shown in Fig. S6b. Thus, although c-di-GMP and c-di-AMP adopt similar extended-bulge conformations, their binding schemes are very distinct, both in the Hoogsteen-edge H-binding, and in the hydrophobic interactions surrounding the purine bases.

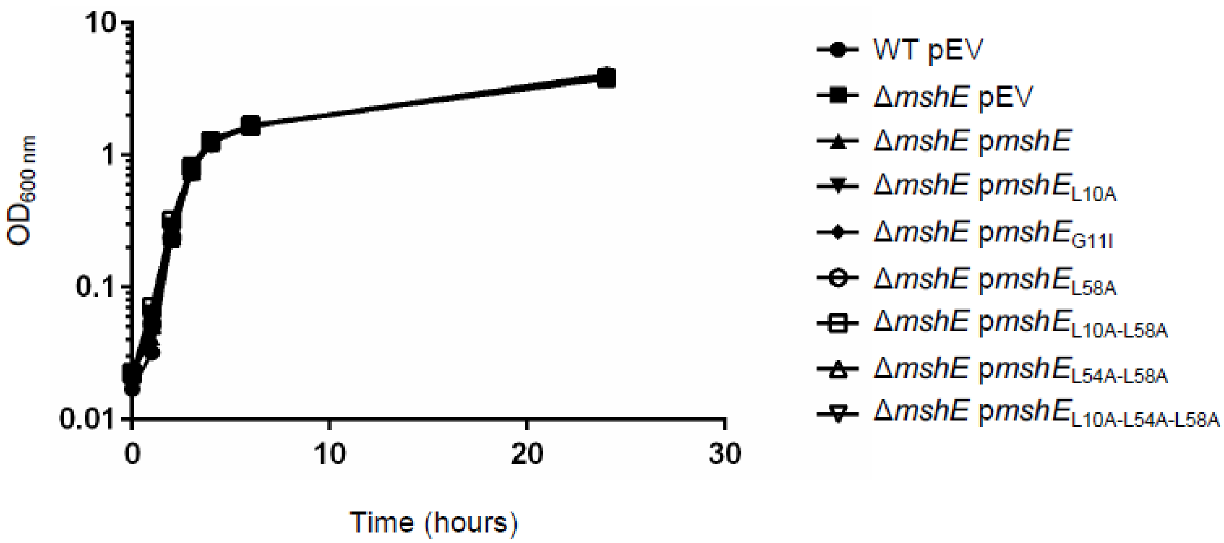

Supplementary Fig. 7: Growth curves of wild type and mutant strains used. Overnight cultures of V. cholerae strains harboring an empty vector (pEV) or constructs with different variants of mshE% were grown on LB (1% typtone%. 0.5% yeast extract, 1% NaCl), pH 7.5 in the presence of ampicillin at 100 μg/ml at 30°C. The cultures were diluted 1:200 in fresh LB broth and Isopropyl β-D-1-thiogalactopyranoside (IPTG) was added to a final concentration of 0.1 mM. The optical density of the cultures (OD600nm) was measured at the intervals indicated in the graph. The graph represents the average OD600nm measurements from two independent biological samples. The error bars represent the standard deviation.

Supplementary Tables

Supplementary Table 1. Cyclic di-GMP binding constants (K_D) of wild-type *Vc*MshEN and its variants determined by ITC titration

*Vc*MshE variant	Ligand/protein ratio (*N*)	Dissociation constant (K_D), M	Fold change
Wild type	0.99	5.0×10^{-7}	1.0
L10A	0.96	1.8×10^{-5}	36.0
L58A	0.81	5.0×10^{-6}	10.0
L10A/L58A	ND[2)]	ND	ND
L29V	0.97	2.0×10^{-6}	4.0
L29A[1)]	0.86	1.4×10^{-5}	28.0
L39A	0.96	1.9×10^{-5}	38.0
Q32A[1)]	0.94	4.5×10^{-6}	9.0
Q61A	0.96	5.9×10^{-6}	11.8
G40I	0.91	3.6×10^{-5}	72.0
G11L	ND	ND	ND

1) Both L29A and Q32A variants were fused with GST to prevent degradation. GST alone showed no c-di-GMP binding.

2) ND: non-detectable by ITC titration

3) Some of these biophysical results are shown in Fig. 3c and 3d. The ITC data showed that: a) a single change of conserved Leu residue to Ala reduced c-di-GMP binding by more than 10 fold (L10A, L58A, L29A, or L39A, Fig. 3c); b) changes of two conserved Leu residues reduced the binding affinity by >100 fold or even completely abolished the c-di-GMP

binding (L10A/L58A); c) the size of hydrophobic side-chain affects the c-di-GMP binding strength of the *Vc*MshEN motif. When the native L29 residue is changed to Val (a side chain that is shorter by ca. 1 Å), the c-di-GMP binding strength is reduced four-fold. When Leu29 is changed to an even shorter Ala residue, the binding strength is reduced 28-fold (cp. WT, L29V, and L29A, Table I); d) changes of the last Gln residues of the motif (Q32A and Q61A) reduced the binding affinity ten-fold; e) the change of the second conserved Gly residue to Leu or Ile also significantly reduced c-di-GMP binding (G11L and G40I, Fig. 3d and, as the extra side chain atoms will cause considerable steric hindrance with the surrounding strong hydrophobic environment (marked by a double-edge yellow arrow in Fig. 3b).

Supplementary Table 2. Melting temperatures Tm (°C) of the native and representative VcMshEN_N variants in the absence or presence of c-di-GMP determined by the DSF method.

*Vc*MshE variant	– c-di-GMP	+ c-di-GMP	Difference
Wild type	61	71	10
G40I	55	56	1
G11L	53	52	-1
L10A	51	56	5
L25A	51	60	9
L25A/L29A	50	59	9
L54A/L58A	46	54	8
L10A/L54A/L58A	49	53	4

Supplementary Table 3. MshEN-containing proteins in some model organisms

**Organism name**	**Protein name and/or locus tag, GenBank accession**[a,b]	
	**Predicted c-di-GMP binding**	**Predicted non-c-di-GMP-binding**
**Proteobacteria**		
Vibrio cholerae O1 El Tor str. N16961	**MshE (VC0405, AAF93578)**	**PilB (VC2424, AAF95567), GspE (VC2732, AAF95872)**
Pseudomonas aeruginosa PAO1	**PulE (PA2677, AAG06065), PA3740 (AAG07127)**[c]	**PilB (PA4526, AAG07914), PulF (PA5210, AAG08595)**
Bdellovibrio bacteriovorus HD100	**Bd2402 (CAE80219)** PilB (Bd1509, CAE79389)	PilB (Bd1596, CAE79474)
Escherichia coli K-12 MG1655	**NfrB (b0569, AAC73670)**[c]	HofB (b0107, AAC73218)
Methylobacillus flagellatus KT	EpsG (Mfla_2024, ABE50291)[c]	
Myxococcus xanthus DK 1622	**PilB (MXAN_5788, ABF89788)**, MXAN_1090 (ABF89262), MXAN_2513 (ABF92256), MXAN_2807 (ABF87190), MXAN_2924 (ABF88591), MXAN_3211 (ABF91223), MXAN_3836 (ABF91355), MXAN_4436 (ABF86923), MXAN_4438 (ABF86742), MXAN_4666 (ABF89796), MXAN_4667 (ABF91352), MXAN_4739 (ABF92523), MXAN_5196 (ABF92996), MXAN_5497 (ABF90396), MXAN_6627 (ABF90093), MXAN_6863 (ABF89935), MXAN_7396 (ABF91318), MXAN_7500 (ABF87845)	**FrgA (MXAN_1130, ABF88426)**[c], PulE (MXAN_2658, ABF85837), MXAN_1145 (ABF88499), MXAN_4627 (ABF89859)
Rhodobacter sphaeroides ATCC 17029	Rsph17029_0522 (ABN75638)	n/a
Salmonella enterica serovar Typhimurium LT2	n/a	HofB (STM0143, AAL19107)
Shewanella oneidensis MR-1	MshE (SO_4109, AAN57083)	PilB (SO_0416, AAN53499)
Xanthomonas campestris ATCC 33913	XCC0678 (AAM39994)	**XpsE (XCC0660, AAM39976)**, PilB (XCC3097, AAM42368), GspE (XCC4088, AAM43309)
Yersinia enterocolitica subsp. *enterocolitica* 8081	n/a	HofC (YE689, CAL10797)

**Other taxa**		
Acaryochloris marina MBIC11017	PilB (AM1_0419, ABW25476), AM1_0779 (ABW25823), AM1_1153 (ABW26190), AM1_1361 (ABW26393), AM1_1657 (ABW26678), AM1_1845 (ABW26865), AM1_2351 (ABW27361), AM1_4892 (ABW29863), AM1_4963 (ABW29934), AM1_6001 (ABW30933), AM1_6196 (ABW31128), AM1_H0057 (ABW33407)	GspE (AM1_2467, ABW27475), PulE (AM1_B0148, ABW31873), PulE (AM1_F0070, ABW33114)
Aquifex aeolicus VF5	n/a	TapB (aq_1971, AAC07748)
Cyanothece sp. ATCC 51142	cce_1044 (ACB50395)	n/a
Deinococcus radiodurans R1	GspE (DR_1964, AAF11514)	n/a
Moorella thermoacetica ATCC 39073	Moth_1554 (ABC19863)	n/a
Nostoc sp. PCC 7120	PulE (all2444, BAB74143)	alr2324 (BAB74023)
(Pepto)clostridium difficile 630	**PilB1 (CD3512, CAJ70416)**	PilB2 (CD3296, CAJ70193)
(Rumini)clostridium thermocellum ATCC 27405	Cthe_1107 (ABN52339)	Cthe_0853 (ABN52087)
Synechocystis sp. PCC 6803	**PilB1 (slr0063, BAA10292**), sll0547 (BAA10486), sll1921 (BAA17267)	**PilB2 (slr0079, BAA10551)**
Thermotoga maritima MSB8	PulE (TM0837, AAD35919)	n/a
Thermus thermophilus HB27	PilF (TTC1622, AAS81964), PulE (TTC1844, AAS82186)	n/a
Thiobacillus denitrificans ATCC 25259	PilB (Tbd_0547, AAZ96500), PilB (Tbd_0752, AAZ96705)	n/a

a Experimentally characterized proteins[3-14] are shown in bold. Predictions of c-di-GMP binding (except for MshE, PA2677, PA3740, and Bd2402) or non-binding (except for PA4526, VC2424, VC2732, XCC0660 and XCC4088) are based on the degree of conservation of the 53-residue motif described in the main text, see Fig. 2a and Supplementary Fig. 3c for representative alignments. PA2677 is 99% identical to the PA14_29490 and PA5210 is 100% identical to PA14_68820, which have been tested for c-di-GMP binding[8]. Both PilB proteins from *Bdellovibrio bacteriovorus* HD100 have been detected in the screen for c-di-GMP binding proteins[10].

b GenBank accessions (in blue) are linked to the respective entries in the NCBI protein database.

c In the bacteriophage N4 adsorption protein NfrB of *E. coli*, which is required for the N4 infection[4], MshEN is associated with a periplasmic glycosyltransferase domain that belongs to the GT2 family and has the typical DxD..Dx_{35}QxxRW active site signature; it probably participates in peptidoglycan remodeling that allows the passage into the host cell of the 320-kDa phage RNA polymerase. Accordingly, many (albeit not all) bacteria that are sensitive to N4-like phages also encode NfrB-like proteins. The *Methylobacillus* sp. protein EpsG, which is involved in the production of the exopolysaccharide methanolan[15], combines the MshEN_N subdomain with an ATPase domain, which, however, is different from the T2SSE ATPase. This protein has been proposed to serve as a chain length determinant protein tyrosine kinase[16]. A number of other MshEN-containing domain architectures are shown in Supplementary Table 4, as well as listed in public domain databases such as Pfam and CDD[1,17]. MshEN domain is often associated with previously uncharacterized domains, for example, in the FrgA protein, which is required for normal swarming and fruiting body formation in *M. xanthus*[18]. In the recently described protein PA3740 from *P. aeruginosa*, which is involved in swarming motility[5], MshEN_N domain is linked to a highly divergent T2SSE_N1 domain. Characterization of MshEN as a c-di-GMP binding regulatory domain will help in characterization of these additional domains and help in understanding the functions of the respective proteins.

Supplementary Table 4. MshEN-containing domain architectures

**Domain organization**[a]	**Representative proteins**[b]	**Phylogenetic distribution**	**Reference**
**MshEN-containing domain architectures**			
MshEN – T2SSE	*Vibrio cholerae* MshE (AAF93578), *Myxococcus xanthus* PilB (MXAN_5788, ABF89788), *Pseudomonas aeruginosa* PilB (PA4526, AAG07914), *Synechocystis* sp. PCC 6803 PilB1 (slr0063, BAA10292) *Xanthomonas campestris* XpsE (XCC0660, AAM39976)	Most bacterial phyla	2, 3, 5, 14, 16, 17, 18
$(MshEN)_2$ – T2SSE	(*Rumini*)*clostridium thermocellum* Cthe_1107 (ABN52339)	Clostridia	
$(MshEN)_3$ – T2SSE	*Deinococcus geothermalis* Dgeo_0623 (ABF44566)	Deinococcus-Thermus, Dictyoglomi	
$(MshEN)_4$ – T2SSE	*Ca.* Magnetobacterium bavaricum MBAV_005423 (KJU82386)	Nitrospiraceae	
MshEN – xT2SSE_N1	*Pseudomonas aeruginosa* PA3740 (AAG07127)	Beta- and gamma-proteobacteria	5
GAF – MshEN – T2SSE	*Geobacter lovleyi* Glov_1971 (ACD95687)	Gamma- and delta-proteobacteria	
REC – MshEN – T2SSE	*Geobacter metallireducens* Gmet_3473 (ABB33681)	Beta-, gamma- and delta-proteobacteria	
MshEN – T2SSE – cNMPbind	*Desulfotalea psychrophila* DP2226 (CAG36955)	Desulfobacterales	
MshEN – HD-GYP – REC – DUF4388	*Myxococcus xanthus* MXAN_2809 (ABF87190)	Myxococcales	
Glyco_trans_2_3 – MshEN	*Escherichia coli* NfrB (AAC73670)	Actinobacteria, Proteobacteria	3,4
MshEN – Glyco_trans_2_3	*Rhodobacter sphaeroides* Rsph17029_0522 (ABN75638)	Actinobacteria, Firmicutes, Proteobacteria	
MshEN – REC	*Geobacter lovleyi* Glov_2262 (ACD95978)	Deltaproteobacteria, Deinococcus-Thermus	
Pkinase – MshEN – UNK – REC	*Stigmatella aurantiaca* PKN11 (STAUR_4477, ADO72257)	Myxococcales	
MshEN – UNK – HEAT	*Myxococcus xanthus* FrgA (MXAN_1130, ABF88426)	Myxococcales	18

**MshEN_N only, no T2SSE_N1 domain**			
$(MshEN\_N)_2$	*Cyanothece* sp. ATCC 51142 cce_1044 (ACB50395)	Cyanobacteria	
REC – MshEN_N – UNK	*Bdellovibrio bacteriovorus* Bd2402 (CAE80219)	Deltaproteobacteria	10
MshEN_N – AAA_31	*Methylobacillus* sp. EpsG (BAC55137)	Beta- and gamma-proteobacteria	15,16
$(MshEN\_N)_2$ – CheX	*Geobacter lovleyi* Glov_2742 (ACD96455)	Deltaproteobacteria, Firmicutes	
MshEN_N – Peptidase_M23	*Acaryochloris marina* NlpD (AM1_1153, ABW26190)	*Acaryochloris, Crinalium*	
$(MshEN\_N)_2$ – REC – ACyc	*Myxococcus xanthus* MXAN_7396 (ABF91318)	Myxococcales	
HPT – CheY-binding – MshEN_N – H-kinase_ dim – HATPase_c – CheW	*Aeromonas veronii* CheA (EKB11881)	Actinobacteria, Proteobacteria	
UNK – TrxB – MshEN_N	*Acaryochloris marina* AM1_1657 (ABW26678)	*Acaryochloris, Pseudanabaena*	

a Domain names are taken from Pfam[1], CDD[17], or explained in the main text. UNK indicates an uncharacterized domain, xT2SSE_N1 is a highly diverged T2SSE_N1 domain. See Supplementary Fig. 3 for representative alignments and http://pfam.xfam.org/family/PF05157#tabview=tab1 for additional domain architectures.

b GenBank accessions (in blue) are hyperlinked to the respective entries in the NCBI protein database. Some of the listed proteins lack a recognizable c-di-GMP-binding motif.

Supplementary Table 5. Some organisms where the MshEN domain is the only known c-di-GMP-specific protein receptor

Phylum, organism name[a,b]	Proteins per genome							c-di-GMP-binding riboswitch
	GGDEF	GGDEF +EAL	EAL	HD-GYP	PilZ	Other	MshEN	
**Actinobacteria**								
Arthrobacter chlorophenolicus A6	1	3	2	-	-	-	1	-
Cellulomonas flavigena DSM 20109	2	6	2	-	-	-	1	2
Eggerthella lenta DSM 2243	9	10	-	2	-	-	2	-
Rubrobacter xylanophilus DSM 9941	2	5	1	3	-	-	1	-
**Aquificae**								
Hydrogenobacter thermophilus TK-6	3	1	3	-	-	-	1	-
**Chloroflexi**								
Anaerolinea thermophila UNI-1	15	3	2	10	-	-	1	-
Chloroflexus aggregans DSM 9485	11	3	-	5	-	-	1	-
Chloroflexus aurantiacus J-10-fl	14	3	-	4	-	-	1	-
Roseiflexus castenholzii DSM 13941	9	3	-	5	-	-	3	-
**Cloacimonetes**								
Ca. Cloacimonas acidaminovorans	2	-	-	2	-	-	1	
**Cyanobacteria**								
Nostoc sp. PCC 7120	8	6	1	2	-	-	1	-
Synechococcus sp. PCC 7002	7	6	2	-	-	-	3	-
Synechocystis sp. PCC 6803	13	9	4	2	-	-	3	-
**Deinococcus/Thermus**								
Deinococcus deserti VCD115	26	11	-	11	-	-	1	3
Deinococcus geothermalis DSM 11300	12	-	-	5	-	-	2	1
Deinococcus radiodurans R1	12	4	1	4	-	-	1	2
Meiothermus ruber DSM 1279	12	3	-	9	-	-	2	1
Thermus thermophilus HB27	7	1	1	5	-	-	2	-
**Dictyoglomi**								
Dictyoglomus thermophilum H-6-12	7	2	-	8	-	-	2	-
Dictyoglomus turgidum DSM 6724	6	2	-	9	-	-	2	-

**Phylum, organism name**[a]	**Proteins per genome**							**C-di-GMP-binding riboswitch**
	**GGDEF**	**GGDEF +EAL**	**EAL**	**HD-GYP**	**PilZ**	**Other**	**MshEN**	
**Firmicutes**								
Clostridium perfringens str. 13	5	1	2	1	-	-	1	2
Halanaerobium hydrogeniformans	25	2	-	22	-	-	2	1
Mahella australiensis 50-1 BON	3	1	4	1	-	-	2	-
Ruminococcus albus 7	30	9	5	6	-	-	2	3
**Fusobacteria**								
Ilyobacter polytropus DSM 2926	16	9	1	8	-	-	2	6
**Gemmatimonadetes**								
Gemmatimonas aurantiaca T-27	8	5	2	7	-	-	2	-
**Planctomycetes**								
Isosphaera pallida ATCC 43644	4	-	-	1	-	-	2	-
**Proteobacteria**								
Paracoccus denitrificans PD1222 (α)	2	-	2	-	-	-	1	-
Rhodobacter capsulatus SB 1003 (α)	2	16	2	-	-	-	1	-
Roseobacter denitrificans OCh 114 (α)	2	6	1	-	-	-	1	-
Ruegeria pomeroyi DSS-3 (α)	4	1	1	-	-	-	1	-
Sulfuricurvum kujiense DSM 16994 (ε)	51	30	5	16	-	-	1	1
Sulfurimonas autotrophica DSM 16294 (ε)	31	21	2	6	-	-	3	1
Xanthobacter autotrophicus Py2 (α)	11	7	-	-	-	-	1	-
**Thermotogae**								
Kosmotoga olearia TBF 19.5.1	9	-	-	14	-	-	1	-
**Verrucomicrobiae**								
Opitutus terrae PB90-1	1	-	-	1	-	-	1	-

[a] - The table includes a selection of organisms that (i) encode at least one GGDEF domain; (ii) do not encode PilZ domains or other known c-di-GMP receptors (BcsE, BldD, FleQ, or VpsT), and (iii) encode at least one MshEN_N domain (with or without the c-di-GMP-binding motif). The number of predicted c-di-GMP-binding riboswitches is taken from Rfam (http://rfam.xfam.org/). For details, see the c-di-GMP census at http://www.ncbi.nlm.nih.gov/Complete_Genomes/c-di-GMP.html.

Supplementary Table 6. Binding constants (K_D) of MshEN_N domains containing proteins with c-di-GMP as determined by ITC titration

Organism, protein (accession, boundaries)	Ligand/ protein ratio	Dissociation constant (K_D, μM)	Reference
**Full-length MshE**			
V. cholerae His-MBP-MshE$_{1-575}$	N/A[d]	1.9[a]	5
Pseudomonas aeruginosa PA14_29490	N/A[d]	0.48[a]	5
**MshEN domain**			
P. aeruginosa PA3740 (AAG07127)[e]	N/A[d]	0.03[b]	18
V. cholerae MshEN$_{1-145}$	0.99	0.5	This work
D. geothermalis PilB_2 (ABF44566$_{151-300}$)	1.01	0.0135	This work
D. geothermalis PilB_3 (ABF44566$_{301-475}$)	N/A[d]	ND[d]	This work
Moorella thermoacetica GspE (ABC19863$_{1-141}$)	1.01	0.46	This work
**MshEN_N domain**			
Bdellovibrio bacteriovorus Bd2402 (CAE80219)[e]	N/A[d]	0.04[c]	29
V. cholerae MshEN_N$_{1-65}$	0.99	0.35	This work
Aeromonas veronii CheA (EKB11881$_{261-390}$)	N/A[d]	2.6	This work

[a] Measured by the DRaCALA method[8]

[b] Measured by solution competition assay[5]

[c] Measured on full-length protein by microscale thermophoresis[10]

[d] N/A, not available; ND, not detectable

[e] In addition to *Vc*MshE, the MshEN-c-di-GMP complex structure presented in this work provides an explanation of the binding mechanism of two recently described c-di-GMP receptors, PA3740 of *P. aeruginosa*[5] and Bd2402 of *Bdellovibrio bacteriovorus*[10]. The PA3740 sequence was initially found using a c-di-GMP specific probe, and an extensive peptide array assay has been used to identify the residues involved in c-di-GMP binding[5]. This led to the identification of the ^{55}LxxxLxxQ^{62} binding motif, which corresponds to the last three conserved residues of our 53-residue-long motif (Fig. 3a). Obviously, many additional residues are required for a high-affinity c-di-GMP binding by PA3740 (K_D ~30 nM[5]). The two-component response regulator Bd2402 has been shown to bind c-di-GMP with K_D of 0.4 μM, although the binding mode has not been characterized[10]. Our analysis showed that Bd2402 contains a MshEN_N subdomain with a well-conserved c-di-GMP binding site (Fig. 2a) downstream of its N-terminal receiver (REC) domain (Supplementary Fig. 3).

Supplementary Table 7. Primers used to subclone the genes used in this study

Name	Sequence(5'-3')	Use
MshE_F1	TACTTCCAATCCAATGCTATGCCGATTAACAAACTGCGT	MshE 5'
MshE_R62	TTATCCACTTCCAATGTTACAGCTGCTGTGCCAGAAAATTC	MshEN_N
MshE_R145	TTATCCACTTCCAATGTTAATAATAGCGGTCAAAACCGTC	MshEN
MshE_R575	TTATCCACTTCCAATGTTACAGATAGATCGGTTCCACCAGAC	MshE 3'
Dgeo_0263_F158	TACTTCCAATCCAATGCTGCCGGTAGCAGCGAAAGCG	Dgeo_0263$_{158-300}$
Dgeo_0263_R300	TTATCCACTTCCAATGTTACGGATAATAACGTTCGATCAGGGCC	Dgeo_0263$_{158-300}$
Dgeo_0263_F327	TACTTCCAATCCAATGCTCAGGCCCGTGGTGGCAAGGT	Dgeo_0263$_{327-471}$
Dgeo_0263_R471	TTATCCACTTCCAATGTCAGCCAAAGTAGCGCTCGATCAGGC	Dgeo_0263$_{327-471}$
Moth_1554_F1	TACTTCCAATCCAATGCTATGGATAGCCGCCGCCGCCT	Moth_1554$_{1-141}$
Moth_1554_R141	TTATCCACTTCCAATGTTACCAAAAGCGGCTCAGGGCTGC	Moth_1554$_{1-141}$
EKB11881_F261	TACTTCCAATCCAATGCTATGAGTCTGCCGGAAGATAG	EKB11881$_{261-395}$
EKB11881_R395	TTATCCACTTCCAATGTTACAGACGCAGGGCGCTAT	EKB11881$_{261-395}$
ACB36052_F269	TACTTCCAATCCAATGCTATGGAACAACAGGGTGAAGC	ACB36052$_{269-400}$
ACB36052_R400	TTATCCACTTCCAATGTTACAGCTGCAGGGCGCTATC	ACB36052$_{269-400}$

Primers used for mutagenesis of the *mshE* gene

Name	Sequence(5'-3')	Use
MshE_L10A_1	CGATTAACAAACTGCGTAAACGCGCGGGCGATCTGCTGGTGGA	MshE$_{L10A}$
MshE_L10A_2	TCCACCAGCAGATCGCCCGCGCGTTTACGCAGTTTGTTAATCG	MshE$_{L10A}$
MshE_G11L_1	CAAACTGCGTAAACGCCTGCTCGATCTGCTGGTGGAAGA	MshE$_{G11L}$
MshE_G11L_2	TCTTCCACCAGCAGATCGAGCAGGCGTTTACGCAGTTTG	MshE$_{G11L}$
MshE_L29A_1	GGCACAGCTGGAACAGGCCGCGAATGCCCAGAAAAATAC	MshE$_{L29A}$
MshE_L29A_2	GTATTTTTCTGGGCATTCGCGGCCTGTTCCAGCTGTGCC	MshE$_{L29A}$
MshE_L29V_1	GGCACAGCTGGAACAGGCCGTGAATGCCCAGAAAAATAC	MshE$_{L29V}$
MshE_L29V_2	GTATTTTTCTGGGCATTCACGGCCTGTTCCAGCTGTGCC	MshE$_{L29V}$
MshE_Q32A_1	GAACAGGCCCTGAATGCCGCGAAAAATACCGGTCGCCG	MshE$_{Q32A}$
MshE_Q32A_2	CGGCGACCGGTATTTTTCGCGGCATTCAGGGCCTGTTC	MshE$_{Q32A}$
MshE_L39A_1	CAGAAAAATACCGGTCGTCGCGCGGGTGATACCCTGATTAGC	MshE$_{L39A}$
MshE_L39A_2	GCTAATCAGGGTATCACCCGCGCGACGACCGGTATTTTTCTG	MshE$_{L39A}$
MshE_G40I_1	AATACCGGTCGCCGCCTGATTGATACCCTGATTAGCCTG	MshE$_{G40I}$
MshE_G40I_2	CAGGCTAATCAGGGTATCAATCAGGCGGCGACCGGTATT	MshE$_{G40I}$
MshE_L54A_1	CTTTCTGAGCGAAACCCAGGCGCTGAATTTTCTGGCACA	MshE$_{L54A}$
MshE_L54A_2	TGTGCCAGAAAATTCAGCGCCTGGGTTTCGCTCAGAAAG	MshE$_{L54A}$
MshE_L58A_1	ACCCAGCTGCTGAATTTTGCGGCACAGCAGCTGAGCCTGC	MshE$_{L58A}$
MshE_L58A_2	GCAGGCTCAGCTGCTGTGCCGCAAAATTCAGCAGCTGGGT	MshE$_{L58A}$
MshE_Q61A_1	CTGAATTTTCTGGCACAGGCGCTGAGCCTGCCGGTGATTG	MshE$_{Q61A}$
MshE_Q61A_2	CAATCACCGGCAGGCTCAGCGCCTGTGCCAGAAAATTCAG	MshE$_{Q61A}$
MshE_L54AL58A_1	CTTTCTGAGCGAAACCCAGGCGCTGAATTTTGCGGCACA	MshE$_{L54A\ L58A}$
MshE_L54AL58A_2	TGTGCCGCAAAATTCAGCGCCTGGGTTTCGCTCAGAAAG	MshE$_{L54A\ L58A}$

Supplementary Table 8. Strains and plasmids used in this study.

Strain or plasmid	Relevant properties	Source
E. coli strains		
DH5-α	F' *endA1 hsdR17 supE44 thi-1 recA1 gyrA96 relA1* Δ(*argF-lacZYA*) U169(Φ80*lac*Δ*M15*)	Promega
S17-1 (λ *pir*)	Tp^r Sm^r *recA, thi, pro,* r_K^- m_K^+ *RP4:2-Tc:*MuKm Tn7 λ *pir*	Ref. [19]
V. cholerae strains		
FY_VC_9573	WT mTn7-gfp, Rif^R Gm^R	Ref. [8]
FY_VC_9586	Δ*mshE* mTn7-gfp, Rif^R Gm^R	Ref. [8]
Plasmids		
pVL393	pMMB derivative with a NdeI site added to the polylinker	Ref. [8]
pVL393-*mshE*	MshE excised from pVL791-*mshE* with NdeI and BamHI and ligated into pVL393 digested with NdeI and BamHI	Ref. [8]
pFY4264	pVL393-*mshE* with point mutation L10A in *mshE*	This work
pFY4265	pVL393-*mshE* with point mutation LG11I in *mshE*	This work
pFY4266	pVL393-*mshE* with point mutation L58A in *mshE*	This work
pFY4267	pVL393-*mshE* with point mutations L10A and L58A in *mshE*	This work
pFY4268	pVL393-*mshE* with point mutations L54A and L58A in *mshE*	This work
pFY4269	pVL393-*mshE* with point mutations L10A, L54A and L58A in *mshE*	This work

Supplementary Table 9: Quantitative analysis of biofilm images.

Strain	WT	ΔmshE							
***mshE* expression**	Empty vector	Empty vector	Wild type	L10A	G11A	L58A	L54/58A	L10/58A	L10/54/58A
**Biomass**	2.230 (0.663)	0.001 (0.002)	1.012 (0.268)	0.021 (0.021)	0.001 (0.001)	2.809 (0.434)	1.705 (0.688)	0.001 (0.001)	1.686 (0.582)
**Significance**		***	*	***	***	ns	ns	***	ns
**Ave. Thickness**	3.332 (1.362)	0.001 (0.001)	1.357 (0.303)	0.564 (0.037)	0.001 (0.001)	3.703 (0.383)	2.559 (0.916)	0.001 (0.001)	2.223 (0.652)
**Significance**		***	**	***	***	ns	ns	***	ns
**Max Thickness**	25.15 (0.33)	4.23 (4.02)	12.53 (2.17)	14.10 (0.81)	5.48 (2.32)	16.76 (0.72)	17.08 (1.18)	4.86 (1.78)	11.75 (2.35)
**Significance**		***	***	***	***	**	**	***	***

Total biomass ($\mu m^3/\mu m^2$), average thickness (μm), and maximum thickness (μm) were calculated using Comstat2. Values presented are means of data from at least three *z*-series image stacks. Significance was determined by an ANOVA. Dunnett's multiple-comparison test identified samples that differ significantly from biofilms formed by the wild-type strain. ns, not significant, * $p \leq 0.05$, ** $p \leq 0.01$, *** $p \leq 0.001$.

Supplementary References

1 Finn, R. *et al.* Pfam: the protein families database. *Nucl. Acids Res.* **42**, D222-D230 (2014).

2 Korotkov, K., Sandkvist, M. & Hol, W. The type II secretion system: biogenesis, molecular architecture and mechanism. *Nat Rev Microbiol.* **10**, 336-351. (2012).

3 Kiino, D. R., Singer, M. S. & Rothman-Denes, L. B. Two overlapping genes encoding membrane proteins required for bacteriophage N4 adsorption. *J. Bacteriol.* **175**, 7081-7085 (1993).

4 Kiino, D. R. & Rothman-Denes, L. B. Genetic analysis of bacteriophage N4 adsorption. *J. Bacteriol.* **171**, 4595-4602 (1989).

5 Düvel, J. *et al.* Application of synthetic peptide arrays to uncover c-di-GMP binding motifs. *J. Bacteriol.* **198**, 138-146 (2016).

6 Nunn, D., Bergman, S. & Lory, S. Products of three accessory genes, *pilB, pilC,* and *pilD*, are required for biogenesis of *Pseudomonas aeruginosa* pili. *J. Bacteriol.* **172**, 2911-2919 (1990).

7 Jones, C. J. *et al.* C-di-GMP regulates motile to sessile transition by modulating MshA pili biogenesis and near-surface motility behavior in *Vibrio cholerae*. *PLoS Pathogens* **11**, e1005068 (2015).

8 Roelofs, K. G. *et al.* Systematic identification of cyclic-di-GMP binding proteins in *Vibrio cholerae* reveals a novel class of cyclic-di-GMP-binding ATPases associated with type II secretion systems. *PLoS Pathogens*, e1005232 (2015).

9 Chen, Y. *et al.* Structure and function of the XpsE N-terminal domain, an essential component of the *Xanthomonas campestris* type II secretion system. *J. Biol. Chem.* **280**, 42356–42363 (2005).

10 Rotem, O. *et al.* An extended cyclic di-GMP network in the predatory bacterium *Bdellovibrio bacteriovorus*. *J. Bacteriol.* **198**, 127-137 (2016).

11 Jakovljevic, V., Leonardy, S., Hoppert, M. & Sogaard-Andersen, L. PilB and PilT are ATPases acting anatagonistically in type IV pilus function in *Myxococcus xanthus*. *J. Bacteriol.* **190**, 2411-2421 (2008).

12 Schuergers, N., Nürnberg, D., Wallner, T., Mullineaux, C. & Wilde, A. PilB localization correlates with the direction of twitching motility in the Cyanobacterium *Synechocystis* sp. PCC 6803. *Microbiology* **161**, 960-966 (2015).

13 Yoshihara, S. *et al.* Mutational analysis of genes involved in pilus structure, motility and transformation competency in the unicellular motile Cyanobacterium *Synechocystis* sp. PCC6803 *Plant Cell Physiology* **42**, 63-73 (2011).

14 Bordeleau, E. *et al.* Cyclic di-GMP riboswitch-regulated type IV pili contribute to aggregation of *Clostridium difficile*. *J. Bacteriol.* **197**, 819-832 (2015).

15 Yoshida, T. *et al.* Genes involved in the synthesis of the exopolysaccharide methanolan by the obligate methylotroph *Methylobacillus* sp strain 12S. *Microbiology* **149**, 431-444 (2003).

16 Ayabe-Chujo, Y., Usami, Y., Yoshida, T., Omori, T. & Nojiri, H. Membrane topology and functional analysis of *Methylobacillus* sp. 12S genes *epsF* and *epsG*, encoding polysaccharide chain-length determining proteins. *Biosci Biotechnol Biochem.* **76**, 608-612 (2012).

17 Marchler-Bauer, A. *et al.* CDD: NCBI's conserved domain database. *Nucl. Acids Res.* **43**, D222-D226 (2015).

18 Cho, K., Treuner-Lange, A., O'Connor, K. A. & Zusman, D. R. Developmental aggregation of *Myxococcus xanthus* requires *frgA*, an *frz*-related gene. *J. Bacteriol.* **182**, 6614-6621 (2000).

19 De Lorenzo, V. & Timmis, K. Analysis and construction of stable phenotypes in gram-negative bacteria with Tn5- and Tn10-derived minitransposons. *Method Enzymol.* **235**, 386-405 (1994).
